# Congenital Insensitivity to Pain and Anhydrosis: Diagnostic and Therapeutic Dilemmas revisited

**DOI:** 10.5005/jp-journals-10005-1288

**Published:** 2015-04-28

**Authors:** KS Ravichandra, Chaitanya Ram Kandregula, Srikanth Koya, Disha Lakhotia

**Affiliations:** Professor and Head, Department of Pedodontics and Preventive Dentistry, Drs Sudha and Nageswara Rao Siddhartha Institute of Health Sciences, Chinnoutpalli, Andhra Pradesh, India; Senior Lecturer, Department of Pedodontics, Drs Sudha and Nageswara Rao Siddhartha Institute of Dental Sciences, Chinnoutpalli Andhra Pradesh, India; Senior Lecturer, Department of Pedodontics, Drs Sudha and Nageswara Rao Siddhartha Institute of Dental Sciences, Chinnoutpalli Andhra Pradesh, India; Senior Lecturer, Department of Pedodontics, Drs Sudha and Nageswara Rao Siddhartha Institute of Dental Sciences, Chinnoutpalli Andhra Pradesh, India

**Keywords:** Congenital insensitivity to pain and anhydrosis, Pain insensitivity, Marvan syndrome, Painless whitlows.

## Abstract

First described in 1932 by Dearborn as ‘congenital pure analgesia’, congenital insensitivity to pain and anhydrosis (CIPA) or hereditary sensory and autonomic neuropathy (HSAN) type IV is an extremely rare autosomal recessive disorder. A 7-year-old female child who is an established case of congenital insensitivity to pain and anhydrosis visited the department of pediatric medicine with osteoarthritic neuropathy. A multidisciplinary team approach was utilized to treat the child under general anesthesia. This article also discusses the diagnostic and therapeutic dilemmas involved in treating this type of children.

**How to cite this article:** Ravichandra KS, Kandregula CR, Koya S, Lakhotia D. Congenital Insensitivity to Pain and Anhydrosis: Diagnostic and Therapeutic Dilemmas revisited. Int J Clin Pediatr Dent 2015;8(1):75-81.

## INTRODUCTION

Congenital insensitivity to pain and anhydrosis (CIPA) or hereditary sensory and autonomic neuropathy (HSAN) type IV is an extremely rare autosomal recessive disorder. Though first described by Leplat in 1846,^1^ not much progression has been made till the turn of the century when Dearborn described the condition as ‘congenital pure analgesia’ in 1932.^2^ Swanson thoroughly studied the condition in 1963^3^ and Mardy first reported the lack of innervations in eccrine sweat glands affecting the patient’s ability to sweat. ‘Painless whitlows’, ‘mal perforant du pied’ and ‘Morvan syndrome’ are some of the many names used to describe a wide range of conditions that are grouped under HSAN. The incidence has been estimated to be one in 25,000 with no predilection toward sex and race.^4^

## ETIOLOGY

The rarity of the condition with only up to 80 cases documented and 300 cases reported in the medical literature throughout the world,^1^ patients not living beyond the age of 25 and the condition sometimes being misdiag-nosed as Lesch-Nyhan syndrome poses difficulty in understanding this type of disorders. However, a lot of work has been done to understand the cause behind the condition. The molecular diagnosis of CIPA has been confirmed for about 80 patients reported in the medical literature (Bonkowsky et al).^5^ A variety of genes are held responsible for the etiology of the condition according to various authors (Table 1).

### Anatomy and Physiology behind the Loss of Pain

Evidence suggests that afferent part of the neural system fails to transmit the pain stimuli to the somatoesthetic cortex.

Two groups have been defined: ‘congenital indifference to pain’ (CIP1) and ‘congenital insensitivity to pain’ (CIP2) which correspond to the two major ascending pathways of pain perception: the ‘medial system’ that project to the anterior cingulated cortex and insula which is associated with the affective response to painful stimuli and the ‘lateral pain system’ that project to the somatosensory cortex provides the sensory discriminative component of pain.^8,9^

**Table 1:** **Table 1:** Various etiologies presented by different authors

*Name of the authors*		*Years*		*The gene responsible*	
Mardy^4^		1999		Neurotrophic tyrosine kinase receptor type 1 (NTRK1) gene, located in chromosome 1	
Shatzky et al^6^		2000		Exons 15 and 16 of the TRKA gene	
Shalimar^7^		2007		Exon 16 (V709L and G718S)	

The term CIP1 describes the individuals with perception of painful stimuli, but with an absence of the affective response; whereas in CIP2, one cannot differentiate the type, intensity and quality of painful stimuli as both the sensory discrimination of pain and the affective response are impaired.^10,11^ In most cases, the condition is a manifestation of HSAN which involves the small-calibre (A-delta and C) nerve fibers, normally transmitters of nociceptive inputs along sensory nerves.^12^

**Table 2:** **Table 2:** Classification of hereditary somatic and autosomal neuropathies (HSAN’s) given by Dyck

*Types of HSAN*		*Features*	
HSAN I		Autosomal dominant neuropathy is characterized by a late onset during the second decade of life; refexes are absent and autonomic involvement is usually limited to reduced sweating and urinary dysfunction. This disease, caused by a mutation in SPTLC1 gene encoding a subunit of serine palmitoyltransferase, determines the loss of all diameters of axons, mainly C and A delta fibers.	
HSAN II		Autosomal recessive neuropathy is characterized by diffuse impairment of discriminative touch and pressure sensation. Also known as ‘Morvan’s syndrome of uncertain cause,’ it has been recently associated with mutations of a novel gene termed HSN2. Bioptic exams show a severe loss of myelinated fibers with relative preservation of unmyelinated one.	
HSAN III		Autosomal recessive neuropathy determines widespread autonomic dysfunction associated with loss of pain and temperature perception. Clinical diagnosis is facilitated by fungiform papillae on the tongue along with an Ashkenazic Jewish ancestry. HSAN III is caused by a mutation in IjB kinase complex associated protein, IKBKAP. Half of patients with this deficiency die before age 30.	
HSAN IV		Extremely rare autosomal recessive disorder associated to diffuse thermal and pain insensitivity, self-mutilation, anhidrosis and recurrent episodes of elevated body temperature with preservation of other sensory modalities. The absence of unmyelinated fibers and losses of small myelinated ones is represented and an association with mutations and polymorphism in the TRKA gene (encoding the receptor tyrosine kynase for nerve growth factor) have been found.	
HSAN V		Onset in childhood with pain and temperature insensitivity is similar to the precedent type except for variable autonomic involvement.	

## CLASSIFICATION

By and large, these conditions can be called as HSANs. However, the wide variation in the clinical findings led researchers to put forward different classifications including that of Pinsky and Digeorge^13^ in 1966. Dyck^14^ in 1984 has popularly classified the neuropathies into five types based on inheritance pattern, time of presentation, clinical and electrophysiological features, metabolic defects and specific genetic markers (Table 2).

## DIAGNOSIS AND IDENTIFICATION

Due to extensive discrepancies in the clinical picture, a variety of diagnostic techniques have been experimented by researchers.

The diagnosis of CIPA usually can be based on the clinical presentation, pharmacological test (intradermic reaction to 1:10,000 histamine) and neuropathological exam in electron microscopy (absence of unmyelinated fibers, reduction in the number of small myelinated fibers, and normal distribution of large myelinated fibers). Detection of mutations on the NTRK1 gene represents as the last diagnostic step.

## HISTAMINE TEST

A histamine test is done by the intradermal injection of 0.05 ml of a 1/10,000 solution of histamine phosphate. Usually, a normal response consists of a local well surrounded by a painful erythematous fare extending for 1 to 3 cm but in a pathological response the fare is absent and the pain minimal.

## SWEAT TEST

A quantitative sweat test can be undertaken in by the application of a gauze pad saturated with 0.4% pilocarpine to the forearm. A low voltage (5mA) electric current canthen be passed between two points 3 cm apart for 5 minutes. Sweat will be collected on blotting paper applied to the area and then sealed in polyethylene for 30 minutes. A normal response is the production of over 80 mg of sweat.^15^

Vasella et al suggested simultaneous injection of acetylocholine with epinephrine which may result in some sweat secretion.^16^

## OTHER TESTS

An electromyography (EMG) can be made to exclude other peripheral neuropathies, and skin biopsy to demonstrate absence of epidermal and sweat gland innerva-tion.^17^ However, Schirmer test to measure lacrimation usually results in a normal response (Swanson 1963). An intradermal injection of histamine phosphate does produce the expected wheal but no axon fare (Pinsky and Di George 1966).^13^

An elevated ratio of homovanillic acid to vanillyman-dellic acid (HMA: V Mi An) in the urine as a diagnosticaid was advocated by Vassela et al.^16^ This finding is similar to that in familial dysautonomia.

## CASE REPORT

**Fig. 1:**
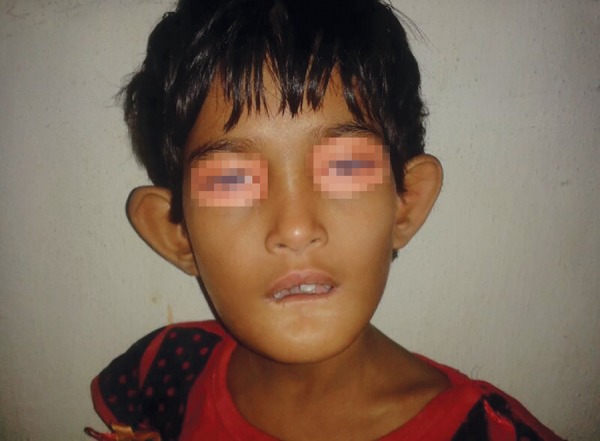
Frontal view of the child

**Fig. 2:**
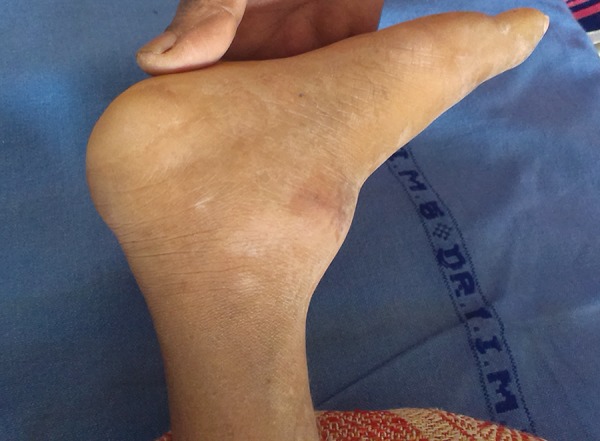
Charcot’s joint

A 7-year-old female child visited the depart ment of pedia-tric medicine with a chief complaint of swelling in the left ankle since 2 weeks (Fig. 1). The patient was already a known case of congenital insensitivity to pain and anhy-drosis. The condition was diagnosed as osteomyelitis of the left ankle (Charcot’s joint, osteoarthritic neuropathy) (Fig. 2) and treatment was initiated for the left ankle. The patient was then referred to the department of pediatric dentistry as she was having bite injuries in the oral cavity.

**Fig. 3:**
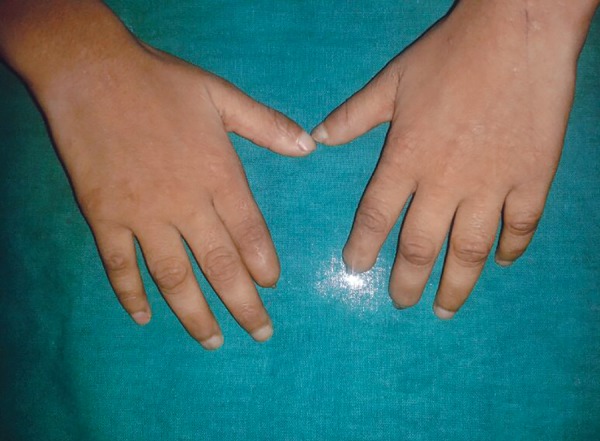
Degenerated nails on the digits of the hands

**Fig. 4:**
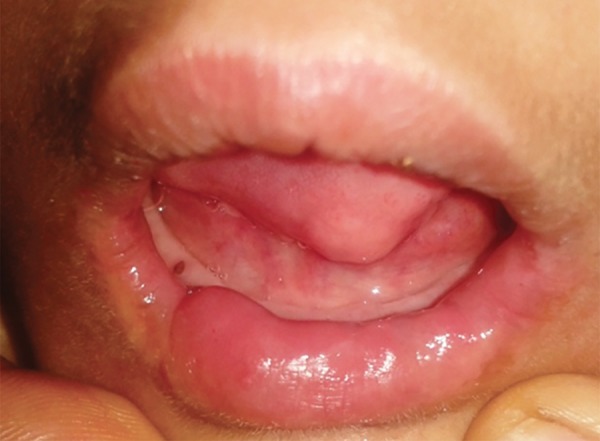
Restricted mouth opening

The family history revealed that the patient was the first child of a consanguineous marriage (first degree relat ives) and had a younger sibling. It was observed that the patient had a waddling gait and subnormal intelligence. The speech development was poor and the child had difficulty in concentrating and understanding. The physical examination disclosed that the patient had dry skin and wounds along with degenerated nails on many of the digits of the hands (Fig. 3). The mouth opening was restricted (27 mm) (Fig. 4). This was her second visit to the dentist.

**Fig. 5:**
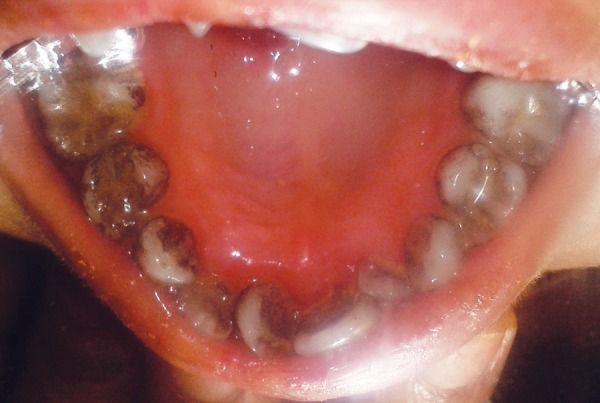
Dental caries in relation to 54 and 64

**Fig. 6:**
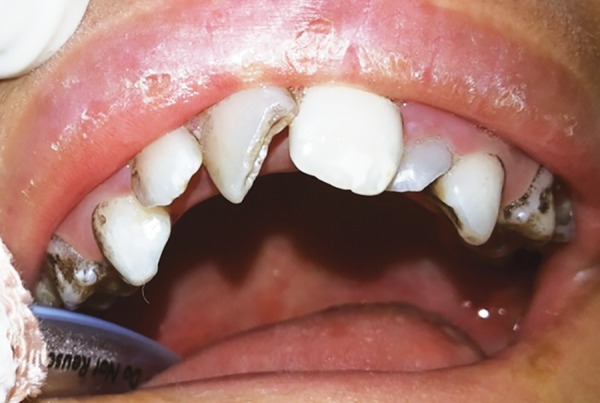
Elli’s class I fracture in relation to 11 and 21

**Fig. 7:**
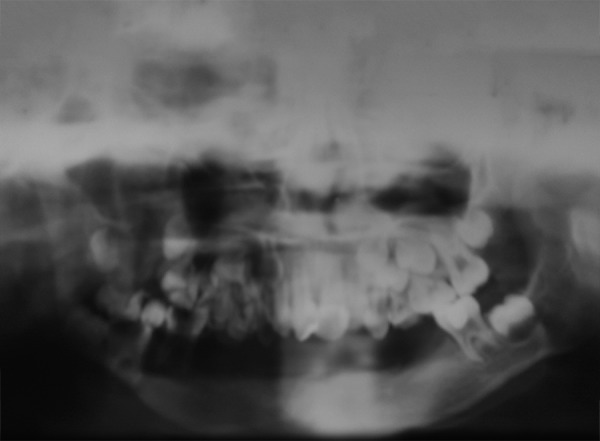
Dental caries involving the pulp in relation to 36

**Fig. 8:**
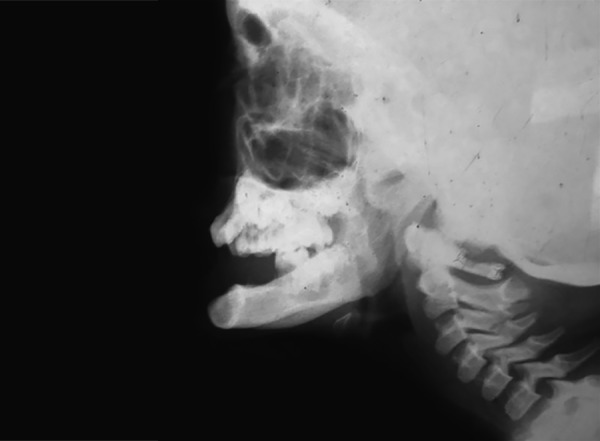
Lateral cephalogram revealing the absence of tooth buds and severe mandibular prognathism

The intraoral examination revealed a soft tissue injury on the tongue and cheeks and she had mixed dentition. The lower arch was edentulous except for the permanent first molars and decay was observed on 54, 64, 36 and 46 (Fig. 5). The maxillary incisors showed Ellis class I fracture (Fig. 6). The orthopantomograph exposed missing permanent tooth buds were and her mother told that the child had the habit of plucking out teeth as soon as they are erupted into the oral cavity due to irritation. This might be attributed to the absence of permanent tooth buds. Also, severe bone loss and dental caries involving the pulp in relation to 36 was noticed (Fig. 7). A lateral cephalogram taken to evaluate the maxillo-mandibular relationship revealed the absence of tooth buds and severe mandibular prognathism (Fig. 8).

**Fig. 9:**
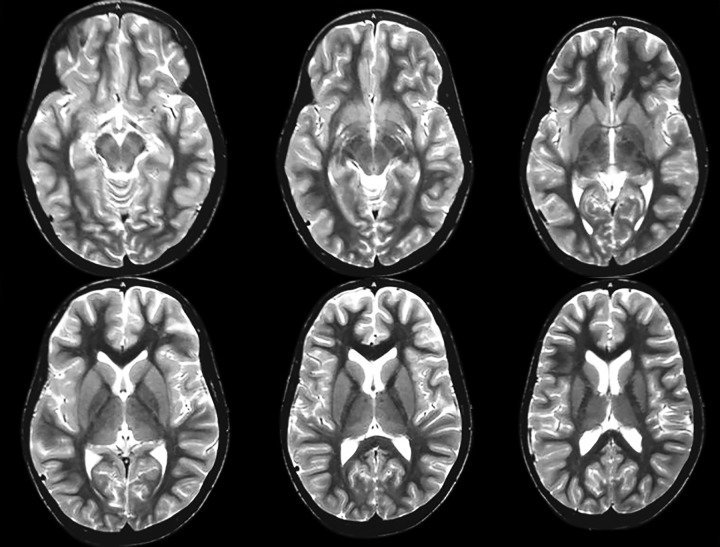
Magnetic resonance imaging revealed no obvious abnormality in the development and the condition of the brain

**Fig. 10:**
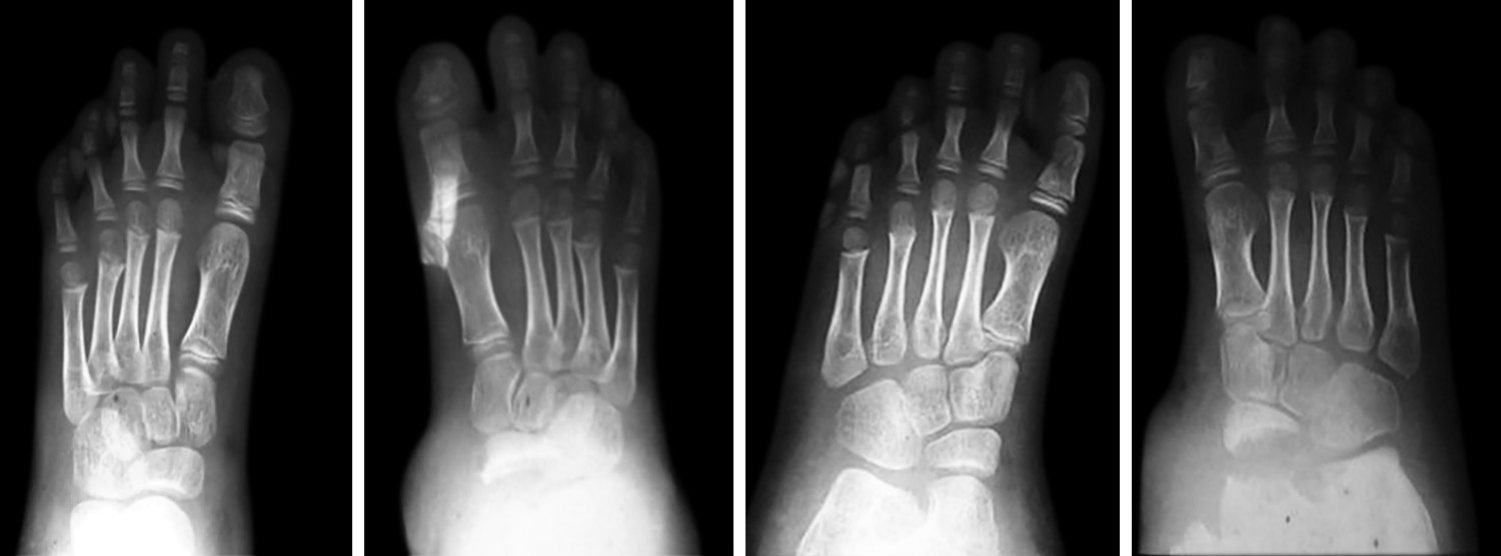
Radiographs of the feet revealed Charcot’s joint

Magnetic resonance imaging was advised by the department of neurology and pediatrics to assess the development and condition of the brain (Fig. 9). The MRI revealed no obvious abnormality in the development and the condition of the brain. Radiographs of the feet revealed Charcot’s joint (Fig. 10).

**Fig. 11:**
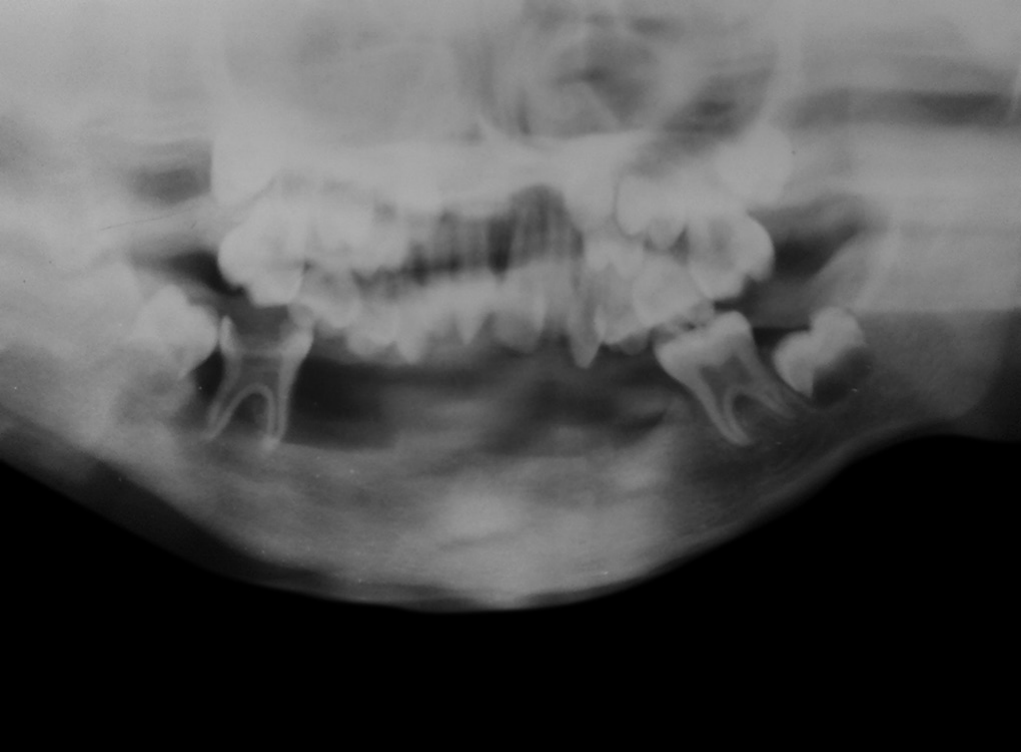
Triple antibiotic paste filled in the pulp chamber and permanent restoration done for 36

**Fig. 12:**
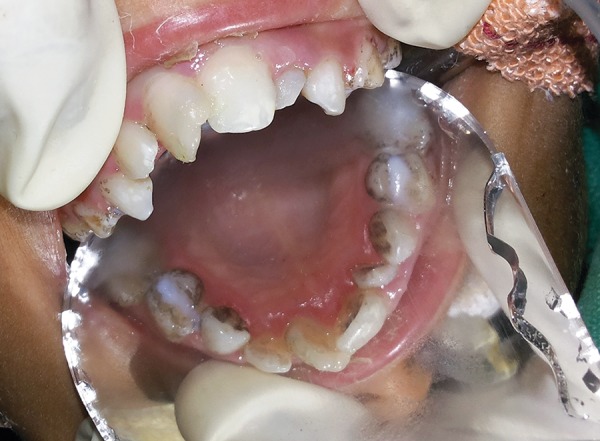
Composite restoration of maxillary central incisors

**Fig. 13:**
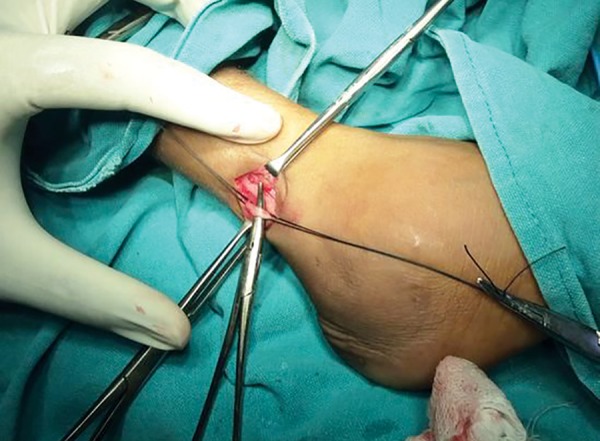
A full thick excision skin biopsy and a nerve biopsy being performed

The treatment was planned under general anesthesia due to the mental condition of the child. A multidisci-plinary approach was planned with the neurology, pediatrics and dermatology departments. Access opening in relation to 36 was done but due to the limited mouth opening negotiating the canals proved impossible. So triple antibiotic paste was filled in the pulp chamber and a permanent restoration was done (Fig. 11). Composite restorations were done for the fractured maxillary incisors (Fig. 12). Simultaneously, a full thick excision skin biopsy and a nerve biopsy were also performed to assess the severity of the condition (Fig. 13).

**Fig. 14:**
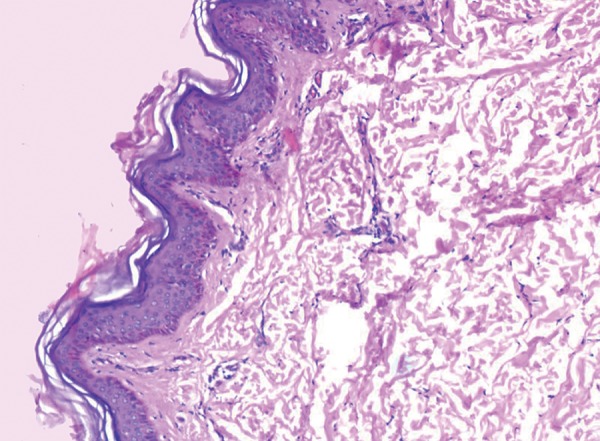
Orthokeratotic squamous epithelium with papillomatosis

**Fig. 15:**
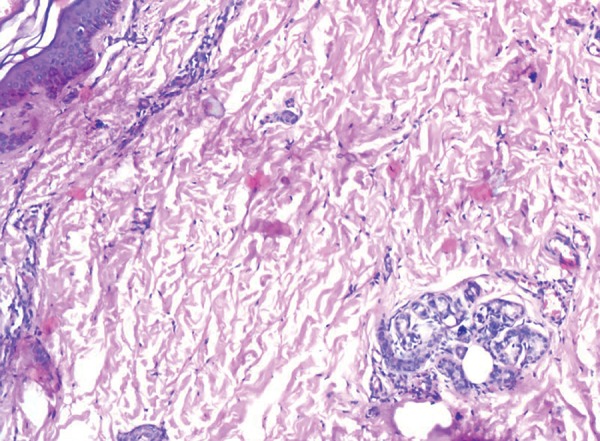
Dermis showing adnexal structures which did not show any nerve fibers

**Fig. 16:**
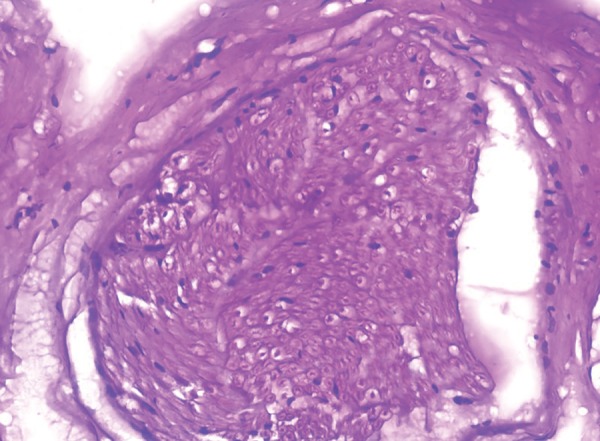
Nerve biopsy showing reduction in the small unmyelinated and myelinated nerve fibers

The skin biopsy revealed orthokeratotic squamous epithelium with papillomatosis (Fig. 14) and dermis showing adnexal structures which did not show any nerve fibres (Fig. 15). Sections from the sural nerve biopsy showed reduction in the small unmyelinated and myeli-nated nerve fibres. However, the large fibres were normal (Fig. 16).

## DISCUSSION

Congenital insensitivity to pain and anhydrosis is widely believed to be mainly caused by a mutation to the TrkA (NTRK1) gene which is responsible for encoding the receptor tyrosine kinase for nerve growth factor. Nerve growth factor is critical for the formation of autonomic neurons and small sensory neurons in the dorsal root ganglia. TrkA is part of the TRK proto-oncogene family and is expressed in neurons that sense temperature and noxious stimuli. A mutation to this gene causes the patient to lose the sensation of pain to noxious stimuli.^18^

Guo studied myelination and axon thickness comparing it to normal myelination in two Taiwanese brothers diagnosed with CIPA. An electromyography was employed to test the motor conduction on the median, ulnar, tibial and perineal nerves followed by sensory conduction of the same nerves. Finally, a sural nerve biopsy was performed and the number of myelinated neurons was counted and axon sizes compared. The tests revealed a loss of small myelinated and unmyelinated fibres but normal large myelinated fibres in the sural nerve. The findings were interesting because it showed that the body tries to cope with less myelinated fibers by increasing the size of the axons. A reduced number of small unmyelinated and myelinated nerves were observed in Sural nerve biopsy of this child which is in agreement with the former study. On the contrary, the increase in the size of axons was not observed.^19^

The deformity known as Charcot joint (neuropathic osteoarthropathy)^19^ was observed in the left ankle due to repeated trauma. Similar finding was reported in other documented literature. One of the major problems in these patients is to differentiate between fractures and infections. Both will usually present at an advanced stage with local swelling and warmth. The prevention of fract ure is thus imperative. Measures, such as custom-fitted shoes and periods of non-weight-bearing may be used to relieve the pressure areas. Education regarding local foot care as well as early medical attention is crucial since these can prevent the call for a radical surgery. The dislocated joints should be treated by ‘watchful neglect’. These joints are painless and mobile.^1^

Primary tooth loss and palmar hyperkeratosis were seen in this child corresponding to the reported case by Bonkowsky et al.^5^ This observation of a premature loss of teeth along with soft tissue injuries may be of great value and dentists should bear in mind that these may be the clinical signs of a rare hereditary disorder.^20^

There are no standard techniques or guidelines prescribed to treat the orofacial self-inficted injuries. Hence, treatment plan is predicted according to the individual clinical circumstances. Prevention of the injuries have been suggested by employing tooth extraction, or through the use of mouth guards and splints and elimination of sharp edges of teeth by the addition of composite.^21^

One more vital point to remember is that the extractions must be performed under local anesthesia to promote hemostasis though there is no pain perception felt by the patient. Also, they were reported incidences in which some patients with CIPA have tactile hyperesthesia and some even complained of pain in the postoperative period for reasons unknown.^22^ The appointments must be short and the treatment carried out in a air conditioned room because of the characteristic hyperthermia due to anhydrosis. A continuous follow-up and close monitoring is necessary as it is hoped that, when the patient gets older, the self-mutilating behavior will improve (Kouvelas et at).^17^ It has been reported that older patients learn not to self-mutilate, even though the sensation of pain is absent. The sense of pain is a precursor for large variety of pathological conditions, but its absence for any reason may lead to adverse and potentially life threatening situations.

## CONCLUSION

Prenatal genetic screening using amniocentesis is the sole accessible option to prevent the birth of an affected child as no cure is available. Osteoarticular and orthopedic complications of CIPA patients are one of the most frequent problems that warrant an early recognition, timely intervention, treatment and prevention to mitigate the complications.

The most important characteristic of CIP is the self-mutilating behavior that leads the child to oral ulcera-tions on lips, tongue and cheeks, self-extraction of teeth and also finger and hand biting. This unique condition is further complicated by the subpar intelligence of the child along with the other systemic ailments that pose a great challenge to the pediatric dentist. Thorough knowledge of the condition involving a multidisciplinary approach is indispensable for the management of the condition. Formulating a predictable treatment plan is very difficult in these types of patients and it is better to have a realistic feasible approach rather than having an ideal, impractical advance. Preservation of the orofacial structures should be emphasized to improve the quality of life of these individuals.

## References

[1] BAR-On E, Weigl D, Parvari R, Katz K, Weitz R, Steinberg T (2002). Congenital insensitivity to pain orthopaedic manifestations.. J Bone Joint Surg Br.

[2] Sezgin B, Bolgul N, Hamamci N, Açkiran EA, Çelenk S, Ayna B (2010). Congenital insensitivity to pain: a case report with dental implications.. HKJ Paediat (new series).

[3] Swanson AG (1963). Congenital insensitivity to pain with anhidro-sis: a unique syndrome in two male siblings.. Arch Neurol.

[4] Dave N, Sonawane A, Chanolkar S (2007). Hereditary sensory autonomic neuropathy and anaesthesia–a case report.. Ind J Anaesth.

[5] Bonkowsky JL, Johnson J, Carey JC, Smith AG, Swoboda KJ (2003). An infant with primary tooth loss and palmar hyperkeratosis: a novel mutation in the NTRK1 gene causing congenital insensitivity to pain with anhidrosis.. Pediatrics.

[6] Shatzky S, Moses S, Levy J (2000). Congenital insensitivity to pain with anhidrosis (CIPA) in Israeli-Bedouins: genetic heterogenicity, novel mutations in the TRKA/NGF receptor gene, clinical findings, and results of nerve conduction studies.. Am J Med Genet.

[7] Shalimar A, Sharaf I, Wahida IF, Ruszymah BH (2007). Congenital insensitivity to pain with anhydrosis in a Malaysian family: a genetic analysis.. J Orthopaedic Surg.

[8] Jewesbury ECO, Vincken PW, Bruyn GW (1970). Congenital indifference to pain.. Handbook of clinical neurology, Amsterdam.

[9] Nagasako EM, Oaklander AL, Dworkin RH (2003). Congenital insensitivity to pain: an update.. Pain.

[10] McMurray GA (1950). Experimental study of a case of insensitivity to pain.. Arch Neurol Psych.

[11] Treede RD, Kenshalo DR, Gracely RH, Jones AKP (1999). The cortical representation of pain.. Pain.

[12] Ogden TE, Robert F, Carmichael EA (1959). Some sensory syndromes in children: indifference to pain and sensory neuropathy.. J Neurol Neurosurg Psychiatry.

[13] Pinsky L, Di George AM (1966). Congen ital fam ilial sensor y neuropathy with anhidrosis.. J Pediatr.

[14] Dyck PJ, Ohta M, Dyck PJ, Thomas PK, Lambert EH (1975). Neural atrophy and degeneration predominantly affecting peripheral sensory neurons, in Peripheral Neuropathy.

[15] Karimi M, Fallah R (2012). A Case Report of Congenital Insensitivity to Pain and Anhidrosis (CIPA).. Iran J Child Neurol.

[16] Vassela F, Emprich HM, Kraus-Ruppert R, Aufdemaur F, Tonz D (1968). Congenital sensory neuropathy with anhidrosis.. Arch Dis Child.

[17] Kouvelas N, Terzoglou DPC (1989). Congenital insensitivity to pain with anhidrosis: case report.. Pediat Dent.

[18] Neves BG, Roza RT, Castro GF (2009). Traumatic lesions from congenital insensitivity to pain with anhidrosis in a pediatric patient: dental management.. Dent Traumatol.

[19] Rahalkar MD, Rahalkar AM, Joshi SK (2008). Congenital insensiti-vity to pain and anhidrosis.. Ind J Radiol Imaging.

[20] Paduano S, Iodice G, Farella M, Silva R, Michelotti A (2009). Orthodontic treatment and management of limited mouth opening and oral lesions in a patient with congenital in sensitivity to pain: case report.. J Oral Rehabil.

[21] Zamuri Z, Goh KL, Aminuddin CA, Mohamed Azril MA, Shukrimi A (2011). Congenital insensitivity to pain with recurrent septic arthritis of the left knee.. Int Med J Malaysia.

[22] Inouye D Congenital insensitivity to pain with anhydrosis. Accessed google search engine on: 04/01/2015. Available at:. http://hilo.hawaii.edu/academics/hohonu/documents/Vol06x04CongenitalInsensitivitytoPainwithAnhidrosis.pdf..

